# An external perpendicular magnetic field does not influence survival and DNA damage after proton and carbon ion irradiation in human cancer cells

**DOI:** 10.1016/j.zemedi.2021.11.001

**Published:** 2022-01-17

**Authors:** Sylvia Kerschbaum-Gruber, Fatima Padilla-Cabal, Elisabeth Mara, Birgit Lohberger, Dietmar Georg, Hermann Fuchs

**Affiliations:** 1 Department of Radiation Oncology, Medical University of Vienna, Vienna, Austria; 2 MedAustron Ion Therapy Center, Wiener Neustadt, Austria; 3 University of Applied Science, Wiener Neustadt, Austria; 4 Department of Orthopedics and Trauma, Medical University Graz, Graz, Austria

**Keywords:** Magnetic fields, In-vitro, Biological effects, MR guided proton therapy, MR guided particle therapy, MRgPT

## Abstract

**Background and purpose:**

Magnetic field effects on the radiobiological effectiveness during treatment of magnetic resonance (MRI) guided particle therapy are being debated. This study aims at assessing the influence of a perpendicular magnetic field on the biological effects in two human cancer cell lines irradiated with proton or carbon ions.

**Methods and materials:**

In vitro cell irradiations were performed in water inside a perpendicular magnetic field of 0 and 1 T for both protons and carbon ions. Samples were located in the center of a spread-out Bragg peak at 8 cm water equivalent depth with a dose averaged linear energy transfer (LET_d_) of 4.2 or 83.4 keV/μm for protons and carbon ions, respectively. Physical dose levels of 0, 0.5, 1, 2, 4 and 6 Gy were employed. The irradiation field was shifted and laterally enlarged, to compensate for the beam deflection due to the magnetic field and ensure consistent and homogenous irradiations of the flasks. The human cancer cell lines SKMel (Melanoma) and SW1353 (chondrosarcoma) were selected which represent a high and a low (*α*/*β*)_*x*_ ratio cell type. Cell survival curves were generated applying a linear-quadratic curve fit. DNA damage and DNA damage clearance were assessed via γH2AX foci quantification at 1 and 24 h post radiation treatment.

**Results:**

Without a magnetic field, RBE_10_ values of 1.04 ± 0.03 (SW1353) and 1.51 ± 0.06 (SKMel) as well as RBE_80_ values of 0.93 ± 0.15 (SW1353) and 2.28 ± 0.40 (SKMel) were calculated for protons. Carbon treatments yielded RBE_10_ values of 1.68 ± 0.04 (SW1353) and 2.30 ± 0.07 (SKMel) and RBE_80_ values of 2.19 ± 0.24 (SW1353) and 4.06 ± 0.33 (SKMel). For a field strength of *B* = 1 T, RBE_10_ values of 1.06 ± 0.03 (SW1353) and 1.47 ± 0.06 (SKMel) resulted from protons, while RBE_10_ values of 1.70 ± 0.05 (SW1353) and 2.37 ± 0.08 (SKMel) were obtained for carbon ions. RBE_80_ values were calculated to be 1.06 ± 0.12 (SW1353) and 2.33 ± 0.40 (SKMel) following protons and 2.13 ± 0.25 (SW1353) and 4.29 ± 0.35 (SKMel) following carbon treatments. Substantially increased γH2AX foci per nucleus were found in both cell lines 1 h after radiation with both ion species. At the 24 h time point only carbon treated samples of both cell lines showed increased γH2AX levels. The presence of the magnetic field did neither influence the survival parameters of either cell line, nor initial DNA damage and DNA damage clearance.

**Conclusions:**

Applying a perpendicular magnetic field did not influence the cell survival, DNA repair, nor the biological effectiveness of protons or carbon ions in two human cancer cell lines.

## 1 Introduction

Technological advancements in radiation oncology allow for increasingly conformal treatments aiming at maximizing tumor dose while reducing normal tissue dose burden. However, increased conformity necessitates accurate positioning and organ motion management, especially for cases with organs at risk in close proximity to the tumor or a moving tumor target. Due to its increased soft tissue contrast and zero dose burden, the use of magnetic resonance imaging (MRI) is a promising alternative to X-ray based imaging. MRI-guidance was already successfully implemented in photon beam radiotherapy to increase treatment accuracy [1], [2], [3].

Particle therapy, is even more susceptible for changes in anatomy or organ motion [4], [5].

The feasibility of integrating MRI-guidance for particle therapy, in terms of dosimetry, treatment planning and imaging, was recently demonstrated [6], [7], [8], [9].

However, knowing the potential impact of an external magnetic field on the relative biological effectiveness (RBE) during irradiation with proton or carbon ions of charged particles is a prerequisite for further prototype developments. The Lorentz force, due to the additional magnetic field will influence the charged particle tracks from primary as well as secondary particles, potentially effecting their biological effectiveness. At the moment, experimental data addressing this issue is scarce and inconclusive [10], [11], [12], [13], [14], [15], [16].

To the best of our knowledge, three groups investigated biological effects in magnetic fields for protons, while for carbon ions, two groups have performed experiments so far.

For protons and carbon ions, some changes in RBE were observed only when applying a longitudinal magnetic field [12], while for a perpendicular magnetic field no changes were observed.

Almost all previous studies, irradiated samples in air in the proximal part of the beam using only a single energy layer. Specific linear energy transfer (LET) values were obtained by placing material upstream of the samples, resulting in LET values lower than expected within a homogeneous dose region in the target. Only one study used a spread-out Bragg peak (SOBP) for sample irradiation [16].

Most studies relied solely on clonogenic cell survival assays [11], [13], [15], some used data on DNA damage [14] or on cell cycle and treatment specific apoptosis [16]. One study investigated the application of magnetic fields also before and after the irradiation [10].

Whereas radiation induces a variety of DNA damages, double strand breaks (DSB) pose the biggest threat to cell survival and are most difficult to repair. Following irradiation, the histone H2AX is rapidly phosphorylated (γH2AX) near the DSB [17]. Only Nagle et al. also included DNA damage analyses but decided to investigate the survival endpoint with lung adenocarcinoma cells and embryonic kidney cells, whereas DNA damage was assessed in osteosarcoma U2OS cells [14].

In our study, two human cell lines, exhibiting a low and high (*α*/*β*)_*X*_ ratio, respectively, were used for all investigations including survival, initial DNA damage and DNA damage clearance in the absence or presence of a 1 T magnetic field. Additionally, RBE calculations were performed with X-ray reference irradiations.

So far, limited data is available on the behavior in the mixed particle energies and elevated LET levels of a SOBP as encountered during clinical treatments. Consequently, SOBP plans were used to reproduce clinical scenarios rather than using mono-energetic beams.

## 2 Material and methods

### 2.1 Cell cultures

Two human cell lines were chosen, representing in vitro models with a high (*α*/*β*)_*x*_ ratio (chondrosarcoma, SW1353) and a low (*α*/*β*)_*x*_ ratio (melanoma, SKMel). SW1353 (ATCC® HTB-94™, LGC Standards, Middlesex, UK) were cultured in DMEM/F12 (Dulbecco's Modified Eagle Medium: Nutrient Mixture F-12), supplemented with 10% fetal calf serum, 25 mM HEPES and 100 U/ml penicillin and streptomycin. SKMel (ATCC® HTB-72™, LGC Standards, Middlesex, UK) cultivated in MEM (Minimum Essential Medium Eagle), supplemented with 10% fetal calf serum, 25 mM HEPES, 2 mM l-Glutamin and 100 U/ml penicillin and streptomycin (all GIBCO®, Invitrogen, Darmstadt, Germany). Both cell lines were cultured at 37 °C in a humidified atmosphere with 95% air and 5% CO2. Cells were seeded in chamber slide flasks (Nunc™ Lab-Tek™ II Chamber Slide™ System, Thermo Fisher Scientific, Waltham, MA, USA) with plastic slides at 2 × 105 cells per flask 24 h before irradiation. Immediately prior to the irradiation, the chamber slide flasks were filled air-bubble free with the respective non supplemented medium.

### 2.2 Experimental set-up

Cells were irradiated in a horizontal beam line equipped with a clinical nozzle enabling proton and carbon ion irradiations with a field size of 20 × 20 cm^2^ in the clinical available energy ranges from 62 to 252 MeV and 120 to 402 MeV/u, respectively.

A resistive, water cooled, H-shaped dipole magnet (Danfysik, Taastrup, Denmark) with an air gap of 13.5 cm is mounted on a support system allowing easy positioning at the treatment iso-center. It is capable of generating magnetic field strengths of up to 1 T. The magnetic field homogeneity was evaluated by the manufacturer to be better than 0.93% of the target field size in a sphere of 75 mm diameter around the treatment iso-center.

**Figure 1:**
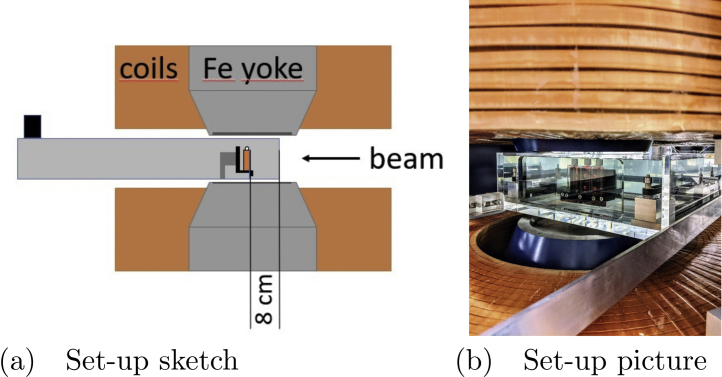
Irradiation set-up. (a) Sketch of the irradiation geometry. The grey box indicates the outline of the water phantom. The cell layer in the flasks (marked orange) is facing the beam. (b) Photo of the actual irradiation set-up inside the magnet. The picture was taken from the front (almost beams eyes view), the slanted view was necessary due to the obstruction caused by the treatment nozzle.

An in-house designed, computer-controlled water phantom [18] was used to position the flasks and detectors within the magnet. A custom holder enabled the positioning of up to 3 chamber flasks in the same depth. An adjustable clamping mechanism allowed to exchange the flasks without additional tools. A dedicated loading position outside the magnet and magnetic field was defined to facilitate flask exchange. The experimental set-up is depicted in Figure 1. Longitudinal as well as lateral position uncertainty of our water phantom was evaluated to be below 0.3 mm. Total positioning errors of the cell layer, including chamber slide variations and set-up variances, were evaluated to be less than 0.6 mm in water equivalent thickness.

### 2.3 Irradiation and dosimetry

For the reference X-ray irradiation, a 200 kV 3 mm Be + 3 mm Al + 0.5 mm Cu filtered YXLON unit (Type TU 32-D03, YXLON GmbH, Hamburg, Germany) was used. X-ray irradiations were performed without magnetic field, using an unrestricted field. The dose rate at the object surface was 1.28 ± 0.02 Gy/min. This value was measured with EBT3 radiochromic films, which were calibrated against a Farmer type ionization chamber in PMMA. Further details about the measurement setup can be found in [19], [20].

Proton and carbon ion treatment plans were created using a research version of the treatment planning system Raystation v7.99 (Raysearch, Stockholm, Sweden), commissioned for our research beam line. Independent dose calculations and dose averaged linear energy transfer (LETd) calculations were performed using the Monte Carlo toolkit GATE RTion v1.0, previously validated by our group for our beam line and magnetic field arrangements [21].

Treatment plans, centered at a water equivalent depth of 80 mm were create for protons and carbon ions, respectively. Both plans covered a target volume of 252 cm^3^ by a SOBP, with lateral, height and depth dimensions of 140 × 90 × 20 mm^3^, respectively. Spot placement and energy layers were selected to ensure a homogeneous dose distribution. At the chamber flask irradiation position of 80 ± 0.6 mm LET_d_ values of 4.1 ± 0.1 and 82.9 ± 3.9 keV/μm were calculated for protons and carbon ions, respectively.

**Figure 2:**
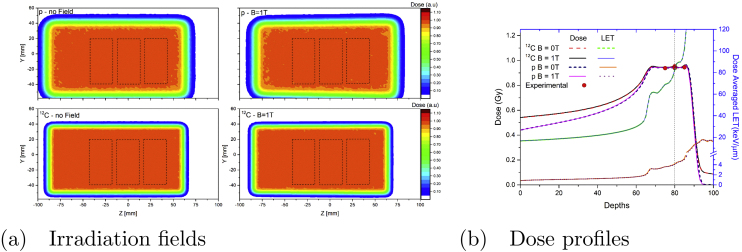
(a): Monte Carlo simulated irradiation field at the measuring depth for protons (top) and 12C (bottom). Chamber flask positions are marked with dashed lines. Results are shown for magnetic field intensities of 0 T (left) and 1 T (right) (b) Dose and LET_d_ profiles for proton and carbon ions, respectively, used for chamber flask irradiation, calculated using the Monte Carlo toolkit GATE. Dose measurements are indicated using red circles. The irradiation position of the chamber flasks located at 80 mm is marked using a vertical dashed line.

Six different physical dose levels of 0, 0.5, 1, 2, 4, and 6 Gy were used for both proton and carbon ion irradiations. Dosimetric verification of the irradiation plans was performed using a Farmer type thimble chamber (PTW-30013, 0.6 cm^3^ active volume, PTW, Freiburg, Germany) in water. Within ±5 mm around the center of the SOBP, dose agreement was always within 1.7% compared to the center of the SOBP (see Figure 2). Correction factors for the Farmer chamber due to the influence of the transverse magnetic fields were measured previously and applied accordingly [22].

The same treatment plans were applied with and without magnetic field. Beam deflections at the center of the SOBP due to the magnetic field were determined using Monte Carlo simulations to be 16 and 6 mm for protons and carbon ions, respectively. To compensate for beam deflections due to the external magnetic field, the treatment plans were laterally enlarged to ensure homogeneous dose irradiations of the flasks with and without magnetic field, without repositioning (see Figure 2a). Monte Carlo simulations were benchmarked against experimental measurements [21] and used to verify the applicability of the chosen experimental set-up.

### 2.4 Clonogenic survival assay

Three independent iterations of the clonogenic survival assays were performed after proton- or carbon ion treatment with and without the presence of the perpendicular 1 T magnetic field. Cells were harvested immediately after irradiation, diluted with supplemented medium appropriate for the cell line and seeded on 6-well plates in concentrations according to the dose level and ion species. For protons, 250 cells (0 and 0.5 Gy), 500 cells (1 and 2 Gy), 1000 cells (4 Gy) and 2000 cells (6 Gy) were seeded per well. For carbon ions, the cell number for each dose level except 0 Gy was doubled. Following a cell line specific incubation period (5 days for SW1353, 7 days for SKMel), cells were fixed with 96% Methanol and stained with 0.5% crystal violet solution. Colonies of more than 50 cells were regarded as surviving clones.

### 2.5 γH2AX assay

In addition to survival parameters, we investigated the effects of the 1 T perpendicular magnetic field on DNA damage induction and processing. Immunolabelling of γH2AX was used in this study to provide quantitative information on the initial formation of γH2AX foci 1 h after irradiation and the loss of γH2AX foci within a 24 h period as a surrogate marker for efficiency of double strand break (DSB) repair. Three independent experimental iterations were performed. Cells were irradiated with 0 or 4 Gy of physical dose of either protons or carbon ions with or without the presence of the 1 T magnetic field. For immunofluorescence detection of γH2AX foci, cells were fixed with 4% paraformaldehyde overnight. Further steps included permeabilization with 0.1% Triton X and 0.1% SDS in PBS for 6 min at room temperature, blocking with 2% BSA in PBS for 1 h at room temperature, primary antibody incubation (1:100, Merck Millipore) for 30 min at 37 °C and secondary antibody incubation (Rhodamine (TRITC)-conjugated AffiniPure Goat Anti-Mouse IgG 1:400 (Jackson Immuno Research Laboratories, West Grove, PA, USA) for 1 h at room temperature. Slides were covered with VectaShield mounting medium containing DAPI (VectorLaboratories Ltd., Peterborough, UK), coverslipped and analyzed with a Zeiss A2 microscope (Zeiss, Oberkochen, Germany) equipped with the automated Metafer analysis system (MetaSystems, Altlussheim, Germany). The Metafer software preselects the brightest foci in each cell nucleus and further on disregards any signal that does not reach a minimum of 60% of signal intensity in the particular nucleus. The settings recommended by the manufacturer were used. 200 cells per slide were scanned, after which malformed cells were removed, resulting in at least 100 cells per slide for further analysis.

### 2.6 RBE modelling and statistical analysis

The linear-quadratic (LQ) formalism was used for survival curve fitting. Surviving fractions in relation to the plating efficiency of non-irradiated control samples were calculated for each value of the delivered physical dose in Gy for both ion species. The mean and standard deviation result from three independent experiments, i.e. 18 individual values, corresponding to three independent 6-well plate per dose group, irradiated at different time points.

Standard errors result from error propagation. A 1/*σ*-weighted minimum chi-square estimation was applied to the LQ model for survival curve fitting [23]. Both parameters, *α* and *β*, were calculated for both radiation types using the same fitting method. For cells irradiated in ion beams, RBEs were calculated for the physical doses that reduced cell survival to 80% (RBE_80_) or 10% (RBE_10_), respectively.

Analysis of γH2AX foci was performed using one-way analysis of variance (ANOVA) with post hoc Tukey's multiple comparison tests. A *p*-value <0.05 was regarded statistically significant. GraphPad Prism 9.1 (GraphPad Software, Inc.) and Python 3.6 programming language (Python Software Foundation, https://www.python.org/) were used for statistical procedures and the graphical illustration [24], [25].

## 3 Results

The cell survival parameters are summarized in Table 1 for X-rays, proton and carbon ion beams.

**Table 1:** Survival parameters of chondrosarcoma (SW1353) and melanoma (SKMel) cells after X-rays, protons and carbon ions.

Cell line	200 kV X-ray			MF [T]	Ion			
	*α*_*x*_ (Gy^−1^)	*β*_*x*_ (Gy^−2^)	*α*_*x*_/*β*_*x*_ (Gy)		p^+^ RBE_10_	p^+^ RBE_80_	^12^C RBE_10_	^12^C RBE_80_
SW1353	0.30 ± 0.04	0.30 ± 0.01	10.00 ± 4.0	0 T 1 T	1.04 ± 0.03 1.06 ± 0.03	0.93 ± 0.15 1.06 ± 0.12	1.68 ± 0.04 1.70 ± 0.05	2.19 ± 0.24 2.13 ± 0.25
SKMel	0.12 ± 0.01	0.04 ± 0.00	3.0 ± 1.0	0 T 1 T	1.51 ± 0.06 1.47 ± 0.06	2.28 ± 0.40 2.33 ± 0.40	2.30 ± 0.07 2.37 ± 0.08	4.06 ± 0.33 4.29 ± 0.35

RBE_10_ values of 1.04 ± 0.03 and 1.06 ± 0.03 (SW1353), 1.51 ± 0.06 and 1.47 ± 0.06 (SKMel) were calculated for protons without (0 T) and with an applied magnetic field (1 T), respectively. Calculation of RBE_80_ values yielded 0.93 ± 0.15 for protons without a magnetic field and 1.06 ± 0.12 for protons with a magnetic field for SW1353. For SKMel, proton RBE_80_ values were 2.28 ± 0.40 (0 T) and 2.33 ± 0.40 (1 T), respectively.

**Figure 3:**
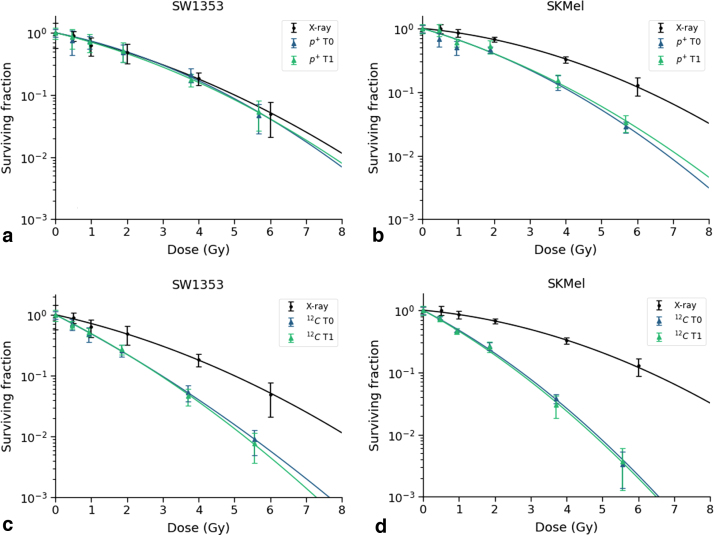
Cell survival after proton or carbon ion irradiation without (0 T) and with an applied magnetic field of 1 T. Cell survival curves of SW1353 and SKMel after reference X-rays (black lines), proton irradiation (a, b) and carbon ion treatment (c,d). Ion irradiations were performed in absence (blue lines) and presence (green lines) of a magnetic field with 1 T field force. Data points represent the mean values of three independent experiments ± standard deviation.

Carbon ion irradiations resulted in RBE_10_ values of 1.68 ± 0.04 without a magnetic field and 1.70 ± 0.05 with a magnetic field for SW1353. For SKMel, RBE_10_ values of 2.30 ± 0.07 and 2.37 ± 0.08 were derived for carbon ion irradiation without and with the magnetic field. RBE_80_ values were calculated to be 2.19 ± 0.24 (0 T) and 2.13 ± 0.25 (1 T) for SW1353 and 4.06 ± 0.33 (0 T) and 4.29 ± 0.35 (1 T) for SKMel, respectively. Survival curves are depicted in Figure 3. Cell survival curves were created for protons and carbon ions with and without a magnetic field. The presence of a magnetic field did not exhibit a statistically significant difference.

**Figure 4:**
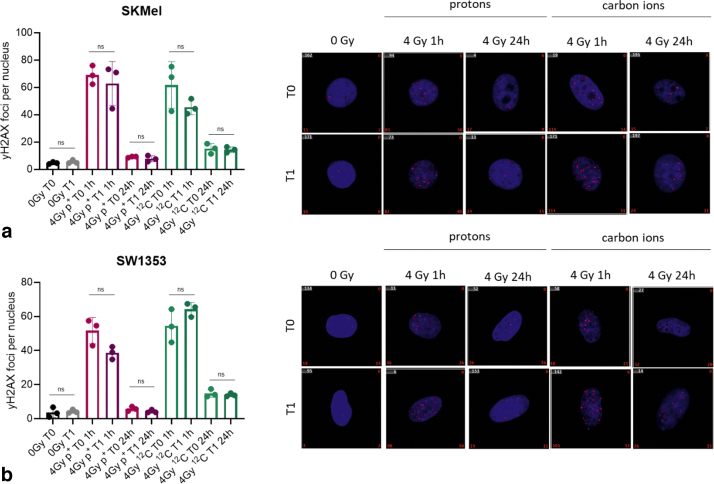
Initial DSB formation or DSB repair within 24 h after proton or carbon ion irradiation of SKMel (a) or SW1353 (b) cells. Quantification and representative im-ages of γH2AX foci (red) in untreated control samples (0 Gy) and after 4 Gy of protons or carbon ions without (0 T) and with (1 T) an additional magnetic field. Nuclei are DAPI (blue) counterstained. Mean values and standard deviations result from a minimum of 3 independent experiments, each containing a minimum of 100 nuclei analyzed. ns = non-significant *p*-value >0.05.

SW1353 (Figure 4a) as well as SKMel (Figure 4b) show substantially elevated amounts of γH2AX foci 1 h post radiation with either ion species. Within 24 h, virtually all proton-induced DSBs were resolved. Carbon ion treatment resulted in higher numbers of residual, unrepaired DSBs 24 h after treatment. Neither cell line demonstrated a difference in initial DSB formation nor for residual DSBs whether an additional magnetic field was applied (see Table 2).

**Table 2:** yH2AX foci in chondrosarcoma (SW1353) and melanoma (SKMel) cells after 4 Gy of protons or carbon ions without (0 T) and with an applied magnetic field of 1 T.

Cell line	MF [T]	4 Gy p^+^				4 Gy ^12^C			
		1 h	*p*-value	24 h	*p*-value	1 h	*p*-value	24 h	p-value
SW1353	0 T	51.7 ± 6.3	0.3421	5.8 ± 1.1	>0.9999	54.4 ± 7.9	0.9993	14.9 ± 1.9	>0.9999
	1 T	38.7 ± 3.0		4.3 ± 0.8		58.1 ± 11.9		14.0 ± 0.8	
SKMel	0 T	69.1 ± 5.6	0.9902	9.1 ± 0.4	>0.9999	61.8 ± 13.9	0.2669	15.2 ± 3.0	>0.9999
	1 T	62.9 ± 13.1		7.8 ± 1.8		45.6 ± 4.5		14.4 ± 1.7	

The influence on the induction or clearance of DNA damage due to the presence of a 1 T magnetic field was not found to be statistically significant.

## 4 Discussion

No significant modification of the survival parameters was found for either cell line following proton and carbon ion irradiations due to the presence of a 1 T perpendicular magnetic field. Moreover, no magnetic field-mediated effects were found for either formation of DNA DSBs or DSB clearance. Differences on initial DNA damage were larger, but not statistically significant. Residual γH2AX foci, indicative of non-repaired and thus challenging DNA DSBs, however showed less variance.

Similar results for cell survival were reported for the low LET_d_ beam entrance regions by others [11], [13], [14], indicating that different LET_d_ values do not influence the magnetic field sensibility.

Interestingly, for protons and carbon ions treated in a longitudinal magnetic field, an improved cell killing was reported for small magnetic field strengths [11], [12]. This effect was found not to be dependent on the magnetic field strength. The source of this difference is unclear and so far, similar effects were not reported by other groups and were also not observed in our study.

The size of the SOBP plateau of 2.5 cm was chosen to optimize irradiation times while providing optimal resilience to positioning errors. The irradiation depth was constrained due to the geometry of our research magnet, but it is still clinically relevant for future MRI-guided particle therapy.

Special focus was put to ensure accurate dosimetry and a repeatable set-up. Dosimetric measurements were cross-checked by Monte Carlo simulations, agreeing with measured data. The chamber response for proton irradiation was previously shown to be affected by magnetic fields [22], requiring a correction factor for absolute dosimetry. Before performing this study, a respective correction factor was determined also for carbon ions using the set-up described above and applied accordingly.

Using the same cell lines and set-up for proton as well as carbon ion irradiation, provides a comprehensive data set on the influence of magnetic fields during particle irradiation. The non-measurable impact of a perpendicular magnetic field on the cell survival as well as on the RBE corroborated results from other groups. Further research with more complex 3D models, normal tissues and eventually animal model may be necessary for the clinical approval and implementation of MR guided particle therapy. Nonetheless, our data show that established methods for RBE calculation/optimization during treatment planning may further be used without additional corrections. It enables the continued development of MR guided particle therapy to bring the advantages of MR guidance to a very conformal treatment technique which potentially will profit even more than conventional MR guided photon therapy.

## 5 Conclusion

The presence of a magnetic field perpendicular to the beam direction during proton and carbon ion irradiation did not lead to significant modification of the survival parameters for SW1353 and SKMel cell lines, nor to initial DNA damage or DNA damage clearance. Relative biological effectiveness remains unchanged within the limits of our experimental uncertainties.

## Conflicts of Interest

The authors declare no conflict of interest
